# Vehicle Position Detection Based on Machine Learning Algorithms in Dynamic Wireless Charging

**DOI:** 10.3390/s24072346

**Published:** 2024-04-07

**Authors:** Milad Behnamfar, Alexander Stevenson, Mohd Tariq, Arif Sarwat

**Affiliations:** Electrical and Computer Engineering Department, Florida International University, Miami, FL 33174, USA; mbehn009@fiu.edu (M.B.); astev028@fiu.edu (A.S.); tmohd@fiu.edu (M.T.)

**Keywords:** decision tree, dynamic wireless charging, gradient boosting, inductive coupler, K-nearest neighbor, random forest, support vector regression, segmented coil array, machine learning, neural network

## Abstract

Dynamic wireless charging (DWC) has emerged as a viable approach to mitigate range anxiety by ensuring continuous and uninterrupted charging for electric vehicles in motion. DWC systems rely on the length of the transmitter, which can be categorized into long-track transmitters and segmented coil arrays. The segmented coil array, favored for its heightened efficiency and reduced electromagnetic interference, stands out as the preferred option. However, in such DWC systems, the need arises to detect the vehicle’s position, specifically to activate the transmitter coils aligned with the receiver pad and de-energize uncoupled transmitter coils. This paper introduces various machine learning algorithms for precise vehicle position determination, accommodating diverse ground clearances of electric vehicles and various speeds. Through testing eight different machine learning algorithms and comparing the results, the random forest algorithm emerged as superior, displaying the lowest error in predicting the actual position.

## 1. Introduction

Electric vehicles are progressively gaining popularity as viable alternatives to traditional fossil fuel-powered counterparts, sparking a strategic transformation within the automotive industry toward embracing electric propulsion. This shift is primarily motivated by growing concerns regarding the depletion of fossil resources and the environmental repercussions associated with conventional fuel-powered vehicles. Despite notable advancements in the design of electric vehicle charging infrastructure, a persistent challenge looms large—the prevalence of range anxiety. This enduring issue acts as a formidable barrier, impeding the widespread acceptance of electric vehicles among the general public. In response to the aforementioned challenges, dynamic wireless charging (DWC) has emerged as a promising technological solution. DWC not only addresses the concerns related to range anxiety but also holds the potential to alleviate the necessity for larger battery capacities in mobile electric vehicles. This groundbreaking technology ensures a reliable and uninterrupted charging experience for electric vehicles in motion. As electric vehicles continue to gain traction as a sustainable mode of transportation, the significance of DWC becomes increasingly evident in mitigating challenges and fostering the widespread acceptance of these innovative vehicles [1].

When exploring the diverse realm of dynamic wireless charging, a pivotal distinction arises based on the length of the transmitter’s coil, leading to the categorization of two primary types: the long-track transmitter and the segmented coil array [2]. The long-track transmitter, as the name suggests, implements an extended transmitter track, enabling the simultaneous charging of multiple vehicles over considerable distances. This design, while promising in facilitating long-distance charging scenarios, comes with inherent drawbacks. Notably, it grapples with challenges such as reduced efficiency and heightened electromagnetic interference. The extended track introduces complexities that impact the overall performance of this charging method. In contrast, the segmented coil array takes a more intricate approach to overcome the limitations associated with the long-track transmitter. This alternative strategy involves the use of multiple transmitter coils, each precisely sized to match the receiver. As the receiver advances along the transmitter track, these coils are activated selectively. This thoughtful activation ensures a targeted and efficient energy transfer, effectively mitigating the efficiency and electromagnetic interference issues encountered with the long-track transmitter design [3].

As the receiver pad travels along the transmitter track, it selectively picks up energy only from the transmitters that are coupled with it. Nonetheless, the high-frequency current in each uncoupled transmitter coil not only induces increased losses and decreased efficiency but also generates problems with electromagnetic interference (EMI). Consequently, it is essential to turn off the uncoupled transmitters, as these inactive transmitter coils are not transferring power to the receiver pad. This control mechanism is referred to as segmentation control [4]. Segmentation control necessitates knowledge of the vehicle’s position to appropriately turn on and off each transmitter coil. Accurate positioning detection is essential to ensure efficient power transfer and precise manipulation of the electromagnetic field [5]. Scholars have extensively investigated receiver position identification, primarily categorized into three approaches: utilizing additional sensors, deploying extra coils for detection, and leveraging circuit parameters of transmitter coils.

Conventional methods for locating and detecting the receiver involve the use of optical, ultrasonic, and magnetic sensors [6]. While coil positioning can be achieved using radio-frequency detection and optic/ultrasound sensors, their effectiveness may be impacted by the magnetic field of the DWC system, necessitating the use of extra hardware [7]. However, sensors are significantly affected by environmental factors, such as rain, shadows, or dirt, leading to inaccurate judgments, especially in rapid switching processes among multiple charging units [8]. Moreover, the inclusion of additional magnetic sensors in these methods results in an elevated cost for the DWC system, and the installation of these sensors adds complexity to the overall DWC system.

Another strategy for detecting the vehicle’s position is the deployment of auxiliary coils. Auxiliary detection coils can be deployed on the primary side [9] or the secondary side [10] of the DWC system to detect the position by identifying changes in magnetic flux. Several additional coils were deployed in [11] to determine the position of electric vehicles. Moreover, to enhance the accuracy of receiver position detection, additional detection coils were strategically placed on both the primary and secondary sides of the DWC system [12]. The typical configuration of this system includes an extra coil for sensing and an identification circuit. The detection circuit transforms the induced voltage from the sensor coil into a signal that the main controller can interpret. The authors of [13] investigated four diverse layouts of detection coils, featuring configurations classified into single-coil, double-coil, and three-coil structures. In [10], an innovative receiver position identification system was employed, which utilized a ferrite core counter system with a Ferrite Position Identification (FPID) group messaging unit, to determine the receiver’s position through the counting of strategically placed ferrite cores within the DWC system. The authors of [14] proposed a dual-side magnetic integration of the inductor of the LCC-LCC compensation network to detect the vehicle position by utilizing a solenoid-integrated coil at the transmitter and a bipolar-integrated coil at the receiver, thereby eliminating the need for extra sensors. However, incorporating additional detection coils in the aforementioned approaches presents a challenge, as it causes an increase in the volume and cost of the DWC system. Furthermore, some of the aforementioned methods are constrained to detecting the receiver’s position in a single direction rather than across a full plane.

The third approach for identifying the vehicle’s position involves leveraging the circuit parameters of the transmitter coils. This method is preferred for detecting the position of electric vehicles as it eliminates the need for additional sensors or coils for detection. The authors of [15] identified the vehicle’s position by measuring the input impedance observed from the first resonator of the array. In [16], an approach was developed for position detection based on measuring the angle of the primary current, which varies depending on the vehicle’s position. Li et al. developed a vehicle position detection method in [17] based on monitoring the primary current, as the magnitude and phase of the current in the transmitter vary according to the receiver’s position, enabling position detection through the calculation of these current characteristics. However, these methods entail the need for multiple current samplings, and determining the RX position relies on the comparison of current values, resulting in a relatively slow response speed.

Machine learning stands out as a highly effective methodology for the prediction and detection of vehicle positions, primarily owing to its inherent self-learning capabilities, adaptability to diverse environments, and rapid response mechanisms. In the work presented by Shen et al. [18], machine learning algorithms were strategically utilized to enhance the adaptability and speed of response in estimating the position of the receiver. Nevertheless, it is imperative to assess the efficiency of machine learning algorithms within the study. This evaluation entails a comprehensive examination of system efficiency, which, in turn, necessitates the meticulous measurement or calculation of parasitic losses occurring in both inductors and capacitors. It is important to note that such measurements may be susceptible to inaccuracies, introducing a layer of complexity to the overall analysis.

The current literature lacks studies considering the influence of speed on predicting the vehicle’s position. Furthermore, there is a noticeable gap in research employing a diverse set of machine learning algorithms for vehicle detection. This absence of comprehensive studies serves as the driving force behind the authors’ motivation to present this paper. In this research, the authors employ various machine learning algorithms for vehicle position detection, providing a novel contribution to the existing body of knowledge. This study meticulously compares results in terms of accuracy and minimum error, shedding light on the performance disparities among the utilized algorithms. To ensure real-world relevance, this investigation incorporates different ground clearances of electric vehicles (air gap) and explores various vehicle speeds, contributing to a more representative analysis. Key input features considered in this study include transmitter current, ΔC (change in current), pad number, air gap, and speeds. This holistic approach aims to capture the multifaceted nature of real-world scenarios and provide insights into the algorithms’ adaptability across diverse conditions. The machine learning algorithms employed encompass gradient boosting, decision tree, support vector regression (SVR), random forest, neural network, K-nearest neighbor (KNN), and Bayesian ridge, each offering distinct advantages and nuances in addressing the complexities of vehicle position detection. The distinctive contributions of this paper, setting it apart from other studies, are as follows:

1. The methodology employed in this research encompasses the strategic utilization of an extensive array consisting of eight distinct machine learning algorithms. The primary objective is to predict the precise position of the vehicle, and this involves conducting a meticulous and comprehensive comparison of the results obtained using each algorithm. The overarching goal is to discern and isolate the most effective algorithm among the diverse set, thereby enhancing the accuracy and reliability of the predictive model.
2. An additional layer of depth is infused into this study through the deliberate inclusion of critical parameters that wield considerable influence in real-world scenarios. Noteworthy considerations encompass the dynamic variability in ground clearances exhibited by different vehicles and the range of speeds at which these vehicles operate. By incorporating these factors, this study endeavors to create a simulation environment that mirrors the complexities inherent in practical situations, thus fortifying the relevance and applicability of the findings.
3. The results for each algorithm are meticulously presented, encompassing detailed regression plots, a thorough analysis of errors specific to each position, and a robust evaluation of the mean squared error. This comprehensive approach is intentionally crafted to foster a nuanced and thorough understanding of the performance intricacies exhibited by each algorithm. The goal is to move beyond mere numerical outputs, offering a comprehensive insight that helps clarify the strengths and limitations of each algorithm in the specific context of predicting vehicle positions.

## 2. Structure of the DWC System

**Figure 1:**
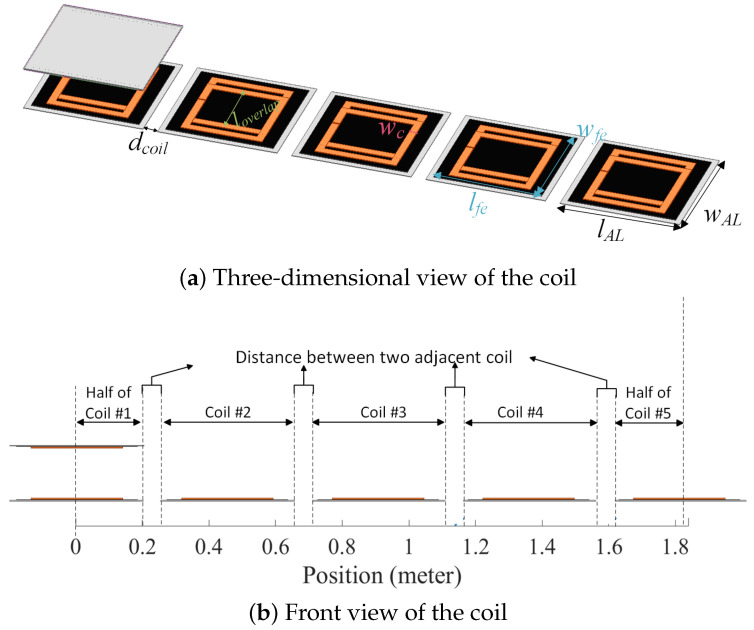
Structure of the coil.

Figure 1 illustrates the structure of the segmented coil array employed in the case study. The transmitter track features five bipolar coils, each the same size as the receiver pad. Table 1 displays the dimensions of the receiver pad and transmitter coils.

When the vehicle aligns with the transmitter coil, the activation of the corresponding transmitter coil becomes essential. Upon the vehicle’s departure, the same transmitter coil should be promptly deactivated, ensuring optimal energy consumption. This strategic activation and deactivation process, synchronized with the vehicle’s presence, not only conserves energy but also minimizes electromagnetic interference (EMI). By selectively activating transmitter coils only within the coverage range of the receiver coil, the dynamic wireless charging (DWC) system achieves significant energy savings. Simultaneously, keeping non-relevant coils inactive contributes to a reduction in EMI, enhancing the overall efficiency and safety of the DWC system. The intricate dance of activation and deactivation is orchestrated by a sophisticated switching control system, reliant on precise information about the vehicle’s position. In essence, this mechanism ensures a harmonious interplay between the vehicle and the charging infrastructure, optimizing both energy utilization and electromagnetic compatibility.

**Figure 2:**
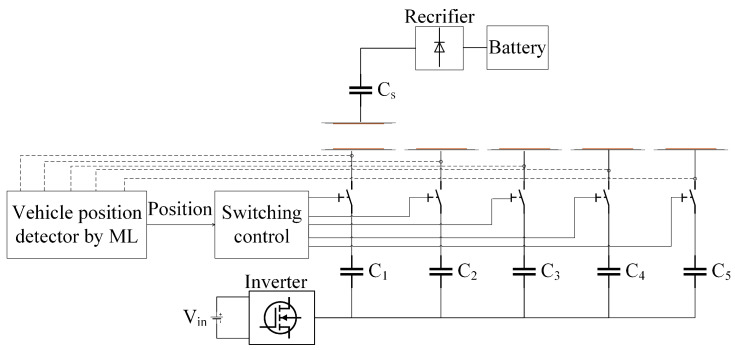
The structure of activating transmitter coils based on machine learning (ML) algorithms.

The diagram in Figure 2 elucidates the mechanism for activating transmitter coils through the integration of advanced machine learning (ML) algorithms. In this setup, each transmitter coil is intricately linked to a dedicated compensation network via individual switches. The orchestration of these switches falls under the purview of a sophisticated switching control unit. This control unit operates the switches in alignment with the vehicle’s position, a determination facilitated by a robust machine learning (ML) algorithm. This study employs a diverse set of machine learning algorithms to enhance the precision of vehicle position identification. The algorithms employed include random forest, decision tree, gradient boosting, support vector regression (SVR), neural network, K-nearest neighbor (KNN), and Bayesian ridge. Each algorithm brings its unique capabilities to the forefront, contributing to the comprehensive evaluation of their effectiveness in optimizing transmitter coil activation based on vehicle position.

After accurately determining the vehicle’s real-time position and mapping the locations of individual transmitter pads, we can optimize coil activation using a lookup table. This table serves to activate specific transmitter coils precisely as the vehicle approaches, ensuring an efficient and responsive dynamic wireless charging (DWC) system.

## 3. Estimation of Vehicle’s Position Using Different Machine Learning Algorithms

In this section, we present the various machine learning algorithms used for estimating the vehicle’s position, considering different air gaps and speeds, and sampling the transmitter current. We can formulate the relationship between the input and output of the machine learning algorithms as follows:

(1) $$y=f\left(x\right)=f\left(d,V,i_{p}\right)$$

where *x* and *y* represent the input and output, respectively, with *y* denoting the position. The input *x* comprises *d*, *V*, and $i_{p}$, representing the air gap, speed, and primary current, respectively. The input *x* can be written as follows:

(2) $$x=\left[\begin{array}{c} x_{1}\\ x_{2}\\ \vdots \\ x_{t} \end{array}\right]=\left[\begin{array}{ccc} d_{1} & V_{1} & i_{p1}\\ d_{2} & V_{2} & i_{p2}\\ \vdots & \vdots & \vdots \\ d_{n} & V_{n} & i_{pn} \end{array}\right]$$

Table 2 presents the database for the parameters, such as the air gap, speed, and position.

We conducted a simulation in ANSYS Maxwell (ANSYS Electronics Desktop 2023) and obtained mutual inductance data for various air-gap values, as listed in Table 2, corresponding to positions ranging from 0 to 1800mm. Subsequently, MATLAB Simulink was employed to derive primary current data based on the mutual inductance data at different vehicle speed values. Ultimately, a total of 22,200 samples were collected. The sample database was formed as follows:

(3) $$\mathrm{Data}=\left\{\left(x_{1},y_{1}\right),\left(x_{2},y_{2}\right)\ldots \left(x_{i},y_{i}\right),i=1,2,3\ldots n\right\}$$

To significantly enhance the accuracy of obtaining vehicle positions, our emphasis is on creating a variety of machine learning (ML) algorithms for position estimation models. Subsequently, we conduct a thorough comparative analysis to evaluate the precision achieved by these ML algorithms in position estimation.

### 3.1. Random Forest

**Figure 3:**
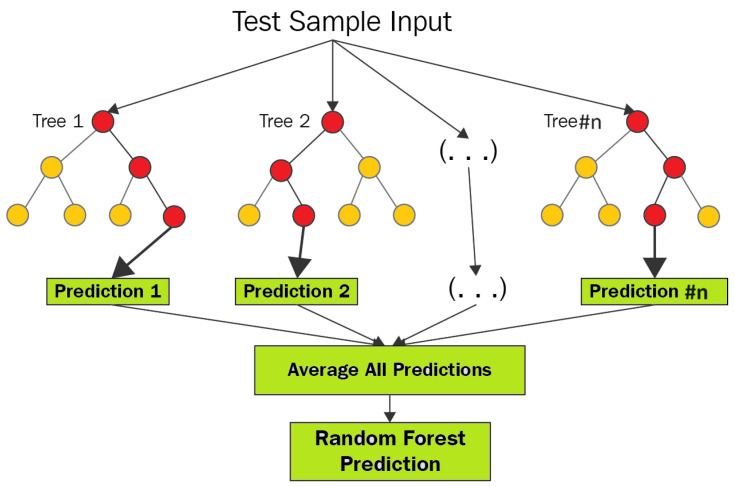
Random forest algorithm structure.

Random forest operates as an ensemble method and is renowned for its effectiveness in addressing classification challenges. Unlike single decision trees, this algorithm trains multiple trees within an ensemble. The collective decision of these trees, based on a majority consensus, determines the final class [19]. Random forest boasts several advantages, including speed, scalability, resilience to noise, and resistance to overfitting. It is user-friendly, eliminating the need for intricate parameter management. Additionally, random forest provides ease of interpretation and visualization. Figure 3 visually represents the structure of the random forest algorithm, showcasing its ensemble-based approach. This methodology ensures versatility and reliability, making the random forest algorithm a robust solution across various classification scenarios.

In the domain of random forests, each tree within the ensemble relies on a set of randomly chosen variables. To formalize this, let $x={\left(x_{1},\ldots ,x_{p}\right)}^{T}$ denote a *p*-dimensional random vector representing the real-valued input or predictor variables, and let *y* be a random variable representing the real-valued response. We assume an unknown joint distribution P(x,y). The main objective is to discover a prediction function f(x) capable of predicting *y*. Ensemble methods construct this prediction function *f* using a set of base learners $h_{1}\left(x\right),\ldots ,h_{J}\left(x\right)$, and these base learners are combined to create the ensemble predictor f(x). In regression tasks, the base learners are typically averaged.

(4) $$\hat{h}\left(x\right)=\bar{y}=\frac{1}{n}\sum_{i=1}^{n}y_{k_{i}};\;\hat{f}\left(x\right)=\frac{1}{j}\sum_{j=1}^{J}{\hat{h}}_{j}\left(x\right)$$

where ${\hat{h}}_{j}\left(x\right)$ is the prediction of the response variable at *x* using the *j*th tree.

Key hyperparameters include the number of trees in the random forest, controlling the maximum depth of each tree to limit the depth, the minimum samples required to split an internal node, and the minimum samples required to be at a leaf node. In the model used, 100 trees were employed with no specified maximum depth, a minimum of two samples were required to split an internal node, and one sample was required to be at a leaf node.

### 3.2. Decision Tree

**Figure 4:**
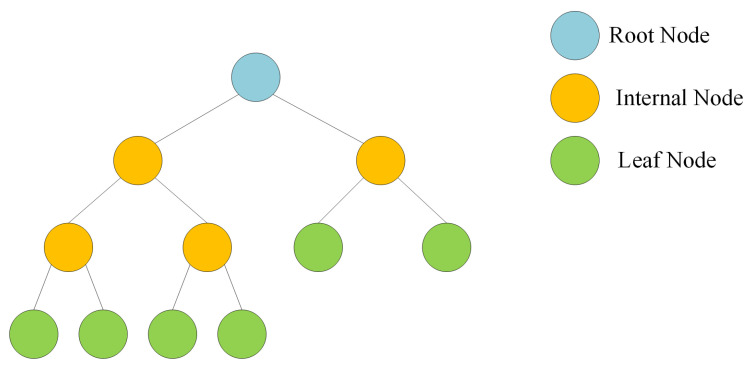
Decision tree algorithm structure.

A decision tree stands out as a supervised machine learning technique specifically designed to tackle both classification and regression problems through a systematic process of data division based on distinct parameters [20]. The leaves of the tree represent the decision outcomes, whereas the nodes facilitate the segmentation of the data. In the context of a classification tree, the decision variable is categorical, leading to binary outcomes like yes/no. Conversely, in a regression tree, the decision variable is continuous, accommodating numerical predictions. The advantages of decision trees are manifold. They exhibit versatility in handling diverse scenarios, whether regression or classification, and provide interpretability. Their effectiveness extends to managing both categorical and quantitative values, along with the ability to handle missing attribute values through imputation. The tree traversal algorithm ensures high performance. However, decision trees face challenges, particularly the risk of overfitting. To overcome this limitation, the random forest technique offers a solution by adopting an ensemble modeling approach [21]. The structure of the decision tree algorithm is visually depicted in Figure 4, emphasizing its hierarchical and branching nature.

The concept of entropy, quantifying the amount of information required for an accurate description of data, is expressed as follows:

(5) $$Entropy\left(S\right)=-\sum_{i=1}^{C}p_{i}\log_{2}\left(p_{i}\right)$$

where *S* is the training dataset, *C* is the number of classes, and *p* is the proportion of *S* classified as *i*. The aim of a split in a tree is to decrease the impurity (uncertainty) in the dataset with respect to the class in the next stage. This objective is achieved by calculating the information gain as follows:

(6) $$Gain\left(S,a\right)=Entropy\left(S\right)-\sum_{v\in \mathrm{Values}}\frac{|S_{v}|}{\left|S\right|}$$

where $S_{v}=s\in S:a(s)=V$, with v being the value of the attribute.

Key hyperparameters include the maximum depth of the tree, regulating the maximum depth, the minimum samples required to split an internal node, specifying the minimum number of samples required to split an internal node, and the minimum samples needed to be at a leaf node. In the model used, there was no specified maximum depth, two samples were required to split an internal node, and a minimum of one sample per leaf node was set.

### 3.3. Gradient Boosting

**Figure 5:**
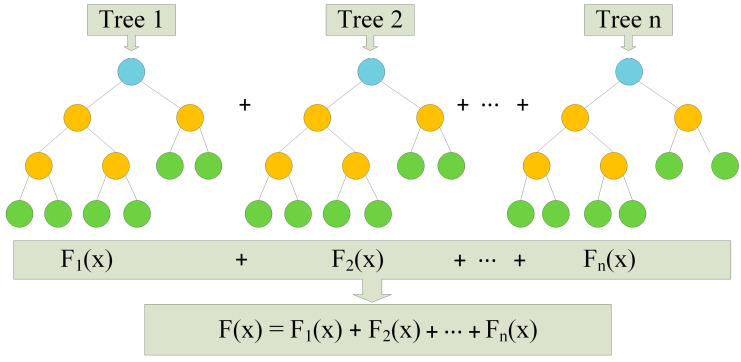
Gradient boosting algorithm structure.

Gradient boosting, a robust algorithm, employs an iterative approach to enhance model performance. It achieves this by fitting a weak learner to the residual errors in each iteration, progressively refining predictions. The strength of gradient boosting lies in its ability to unravel intricate data structures, capture nonlinearity, and detect high-order interactions within the data. This technique proves particularly effective in scenarios featuring a vast number of potential predictors, ranging from hundreds to tens of thousands. As the algorithm iterates, it autonomously refines its understanding of the data, leading to continuous improvements in overall model accuracy [22]. The structural overview of the gradient boosting algorithm is depicted in Figure 5.

In the context of a training dataset $D={\left\{\left(x_{i},y_{i}\right)\right\}}_{i=1}^{N}$, the primary objective of gradient boosting is to derive an approximation, denoted as $\hat{F}\left(x\right)$, for the underlying function $F^{*}\left(x\right)$. This function maps input instances *x* to their corresponding output values *y*. The optimization process involves minimizing the expected value of a predefined loss function L(y,F(x)). Gradient boosting achieves this by constructing an additive approximation of $F^{*}\left(x\right)$ through a weighted sum of functions. Initially, an initial constant approximation of $F^{*}\left(x\right)$ is acquired as follows:

(7) $${\hat{F}}_{0}\left(x\right)=\arg\min_{\gamma }\sum_{i=1}^{N}L\left(y_{i},\gamma \right)$$

Yet, rather than directly addressing the optimization problem, each $h_{m}$ can be interpreted as a greedy step within a gradient descent optimization for $F^{*}$. In this context, every model $h_{m}$ undergoes training on a distinct dataset $D={\left\{\left(x_{i},r_{mi}\right)\right\}}_{i=1}^{N}$, where $r_{mi}$ represents the pseudo-residuals, derived as follows:

(8) $$r_{mi}=-{\left[\frac{\partial L(y_{i},{\hat{F}}_{m-1}\left(x_{i}\right))}{\partial {\hat{F}}_{m-1}\left(x_{i}\right)}\right]}_{\hat{F}\left(x_{i}\right)={\hat{F}}_{m-1}\left(x_{i}\right)}$$

A step size β is chosen as the learning rate, and the model can be updated as follows:

(9) $${\hat{F}}_{m}\left(x\right)={\hat{F}}_{m-1}\left(x\right)+\beta h_{m}\left(x\right)$$

where the final model can be derived as follows:

(10) $$\hat{F}\left(x\right)={\hat{F}}_{M}\left(x\right)$$

Key hyperparameters include the number of boosting stages, the learning rate, controlling the step-size shrinkage, and the maximum depth of the individual trees, which limits the depth. In the model used, 100 boosting stages were applied with a learning rate of 0.1, and the tree depth was limited to three levels.

### 3.4. K-Nearest Neighbor

**Figure 6:**
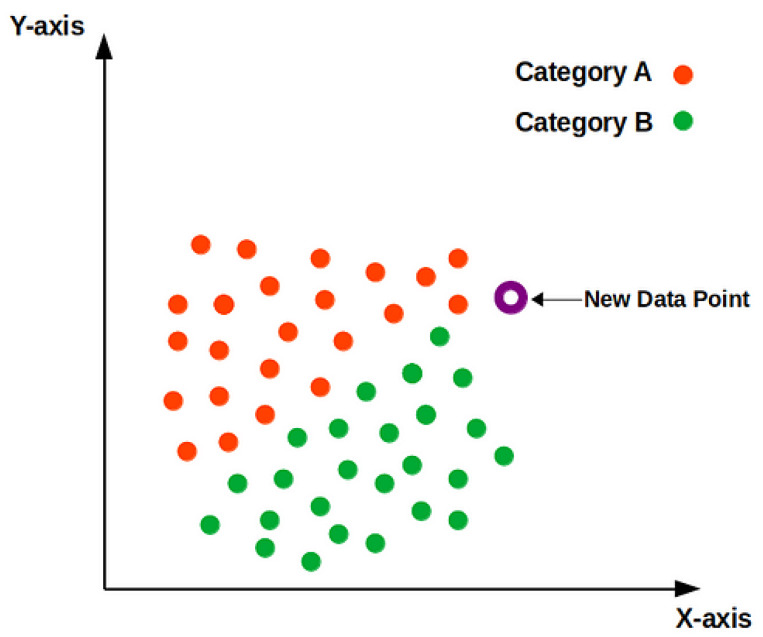
KNN algorithm structure.

Operating as a non-parametric classification algorithm, K-nearest neighbor assigns an unlabeled sample point the class of its nearest neighbor from a set of previously labeled points [21]. This rule operates independently of the joint distribution of sample points and their classifications. Particularly effective for multi-modal classes and scenarios where objects can have multiple labels, it employs a straightforward lazy learning approach, albeit with reduced efficiency. Notably, its performance hinges on the prudent selection of the ‘k’ parameter, with no principled method available except through computationally expensive techniques such as cross-validation. The algorithm is susceptible to the adverse effects of noise and demonstrates sensitivity to irrelevant features. Furthermore, its performance dynamics vary with dataset size, as it necessitates revisiting all data points [23]. Figure 6 shows the structure of the KNN algorithm.

The KNN algorithm employs Euclidean distance metrics for locating the nearest neighbor. The Euclidean distance between $x_{query}$ and each $x_{i}$ in the training set ($D={\left\{\left(x_{i},y_{i}\right)\right\}}_{i=1}^{N}$) is calculated as follows:

(11) $$d_{i}=\sqrt{\sum_{j=1}^{M}{\left(x_{\mathrm{query},j}-x_{i,j}\right)}^{2}}$$

where M is the number of features. The distances $d_{i}$ are sorted in ascending order, maintaining the corresponding indices. Then, the first K indices are selected from the sorted list. These indices correspond to the K-nearest neighbors. Finally, the predicted value for the query point (${\hat{y}}_{query}$) is calculated as follows:

(12) $${\hat{y}}_{\mathrm{query}}=\frac{\sum_{i=1}^{K}\frac{1}{d_{i}}\cdot y_{i}}{\sum_{i=1}^{K}\frac{1}{d_{i}}}$$

The primary hyperparameter is the number of neighbors to consider during prediction. In the model used, KNN was configured with five neighbors, influencing the local smoothing of predictions.

### 3.5. Support Vector Regression

**Figure 7:**
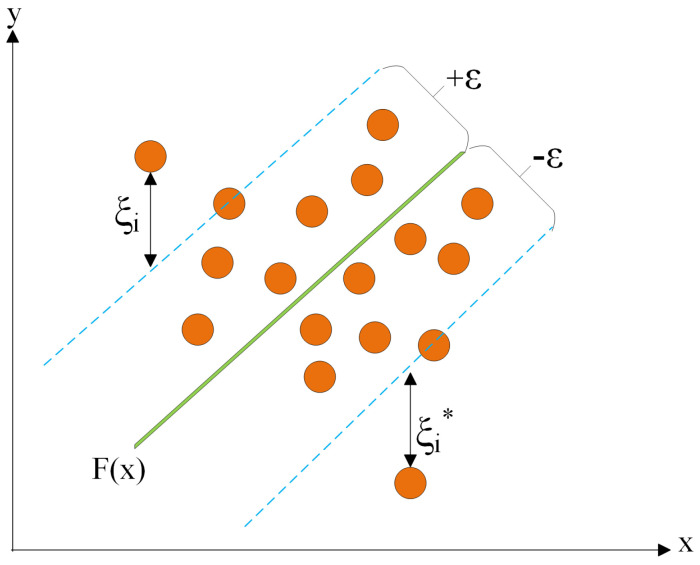
Support vector regression structure.

Support vector regression (SVR) stands out for its utilization of kernels, sparse solutions, and VC (Vapnik–Chervonenkis) control over the margin and the number of support vectors. While not as widely embraced as support vector machine (SVM), SVR has proven effective in the estimation of real-valued functions. Operating as a supervised learning technique, SVR undergoes training with a symmetrical loss function that uniformly penalizes both high and low estimates. A noteworthy advantage of SVR lies in its computational complexity, which remains unaffected by the dimensionality of the input space. Additionally, SVR demonstrates exceptional generalization capabilities, resulting in high prediction accuracy [24]. Figure 7 illustrates the structure of the support vector regression algorithm.

Training dataset $D={\left\{\left(x_{i},y_{i}\right)\right\}}_{i=1}^{N}$, where $x_{i}$ is the input feature vector and $y_{i}$ is the corresponding output. The objective is to find a regression function by solving the following optimization problem:

(13) $$\min_{w,b,\xi ,\xi^{*}}\frac{1}{2}{∥w∥}^{2}+C\sum_{i=1}^{N}\left(\xi_{i}+\xi_{i}^{*}\right)$$

subject to the following constraints:

(14) $$\begin{array}{c} y_{i}-\left(w\cdot x_{i}+b\right)\leq \varepsilon +\xi_{i},\;i=1,\ldots ,N\\ \left(w\cdot x_{i}+b\right)-y_{i}\leq \varepsilon +\xi_{i}^{*},\;i=1,\ldots ,N\\ \xi_{i},\xi_{i}^{*}\geq 0,\;i=1,\ldots ,N \end{array}$$

Given the nonlinear kernel function, the nonlinear SVR model is expressed as follows:

(15) $$f\left(x_{i}\right)=-\sum_{i=1}^{N}\left(\alpha_{i}-\alpha_{i}^{*}\right)x_{i}^{⊤}x_{i}+C\sum_{i=1}^{N}\left(\alpha_{i}-\alpha_{i}^{*}\right),\;\alpha_{i},\alpha_{i}^{*}\in \left[0,C\right]$$

Crucial hyperparameters include the regularization parameter, which determines the regularization strength, and the kernel function, which selects the type of kernel function to be used. In the model used, the regularization parameter was set to 1.0, and the radial basis function kernel was employed.

### 3.6. Neural Networks

**Figure 8:**
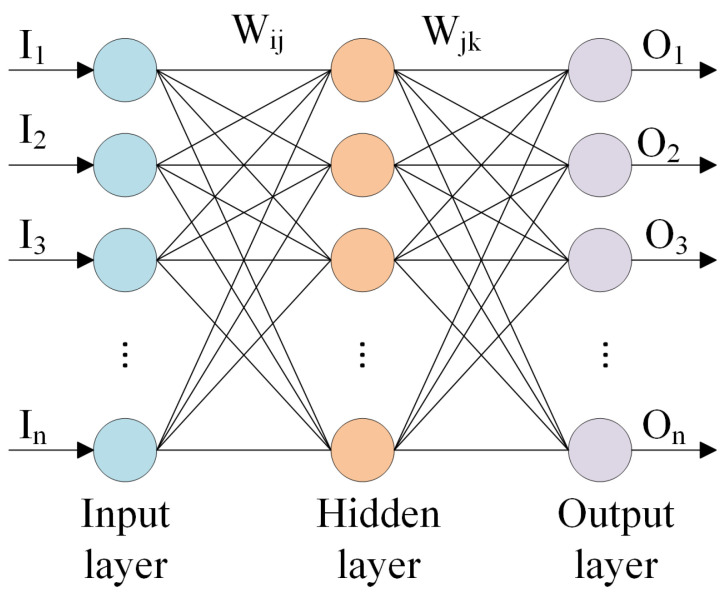
Three-layer backpropagation neural network structure.

Neural networks serve as computational models inspired by the intricacies of the human brain’s structure, processing, and learning mechanisms, albeit on a smaller scale. They excel in handling scenarios where relationships are nonlinear or dynamically evolving. Unlike traditional methods with rigid assumptions, such as normality and linearity, neural networks offer a flexible alternative. Their ability to capture a wide range of relationships enables users to model phenomena that might be challenging or impossible to explain using conventional approaches [25]. Figure 8 shows the structure of a three-layer backpropagation neural network.

The output of the hidden layer can be derived as follows:

(16) $$h_{j}=\sum_{i=1}^{n}W_{ij}I_{i}\;j=1,2,\ldots ,l$$

The output of the output layer can be calculated as follows:

(17) $$O_{k}=\sum_{j=1}^{l}h^{k+1}\left(i\right)W_{jk}\;k=1,2,\ldots ,m$$

Hyperparameters in the sequential model of Keras include the number of units in each dense layer, the number of training epochs, the batch size for optimization, the optimizer algorithm, and the loss function. In the model used, the neural network comprised two hidden layers, each with 10 units and ReLU activation. It was trained for 50 epochs with a batch size of 32, using the ’Adam’ optimizer and optimizing for mean squared error.

### 3.7. Bayesian Ridge

Bayesian ridge regression embraces a probabilistic methodology, leveraging the Gaussian probability distribution. The optimization of posterior predictions in Bayesian regression incorporates the use of l2 regularization. This sets Bayesian ridge regression apart, particularly in the derivation of the weighted coefficient ’w,’ which is deduced from a spherical Gaussian [26].

While Bayesian ridge regression demands computational time, it demonstrates notable adaptability concerning small data parameters. Its user-friendly nature is evident in effectively handling regularization challenges and facilitating the tuning of hyperparameters. Despite the computational demands, Bayesian ridge regression proves to be a valuable tool, especially in scenarios with limited data parameters, offering a practical and efficient approach to regularization problem-solving and hyperparameter fine-tuning.

Let observations $y={\left(y_{1},\ldots ,y_{n}\right)}^{T}\in R^{n}$. Define features $X=\left[1_{n},x_{1},\ldots ,x_{m}\right]\in R^{n\times (m+1)}$, where $x_{i}$ represents the column vectors in $R^{n}$, i=1,…,m, and $1_{n}={\left(1,\ldots ,1\right)}^{T}\in R^{n}$.

We make the assumption that each $y_{i}$ has a likelihood, given by:

(18) $$p\left(y_{i}|\omega ,\alpha \right)=N\left(y_{i}|\sum_{j=1}^{m}\omega_{i}X_{j},\alpha \right)$$

where $\omega ={\left(\omega_{0},\omega_{1},\ldots ,\omega_{m}\right)}^{T}\in R^{m}$ represents the weights and α∈R represents the variance (indicating the noise).

Bayesian ridge regression focuses on determining the “posterior” distribution of the model parameters instead of directly finding these parameters. Consequently, Bayesian ridge regression requires a substantial volume of training data to enhance the accuracy of the model. Key hyperparameters include the values controlling the shape and precision of the distribution of the weights. In the model used, very small values were configured for these hyperparameters, implying weak regularization.

### 3.8. Linear Regression

Linear regression is recognized for its straightforward model structure, representing the regression function as a linear combination of predictors. Its popularity in various applications can be attributed to several factors. The linear form of the model allows for easily interpretable parameters. Furthermore, linear model theories boast well-established mathematical elegance. Additionally, linear regression serves as a foundational element for numerous contemporary modeling tools. Particularly in scenarios where the sample size is limited or the signal strength is modest, linear regression frequently delivers a satisfactory approximation of the underlying regression function [27].

Contemplate the dataset D, denoted as $\left\{\left(y_{i},x_{i}\right):i=1,\ldots ,n\right\}$, where $y_{i}$ represents the ith response measured on a continuous scale, $x_{i}$ is the corresponding predictor vector, and n≫p denotes the sample size. The linear model is formally defined as follows:

(19) $$y_{i}=\beta_{0}+\beta_{1}x_{i1}+\ldots +\beta_{p}x_{ip}+\epsilon_{i}$$

In the form of a matrix, we can write:

(20) y=Xβ+ϵ

where $y={\left[y_{i}\right]}_{n\times 1}$ represents the n-dimensional response vector; $X={\left(x_{ij}\right)}_{n\times (p+1)}$, where $x_{i0}=1$ is commonly referred to as the design matrix; and $\varepsilon ={\left[\varepsilon_{i}\right]}_{n\times 1}$. Models (1) or (2) involve four primary statistical assumptions, which are as follows [27]:

1. Linearity: $\mu \equiv {\left[E\left(y_{i}|x_{i}\right)\right]}_{n\times 1}=X\beta$;
2. Independence: $\varepsilon_{i}$’s are independent of each other;
3. Homoscedasticity: $\varepsilon_{i}$’s exhibit equal variance $\sigma^{2}$;
4. Normality: $\varepsilon_{i}$’s follow a normal distribution.

It is essential to note that many properties of linear models hold true even when all four assumptions are not met. Linear Regression does not have many hyperparameters to tune. It relies on the linear relationship between the features and the target variable.

## 4. Results and Discussion

**Figure 9:**
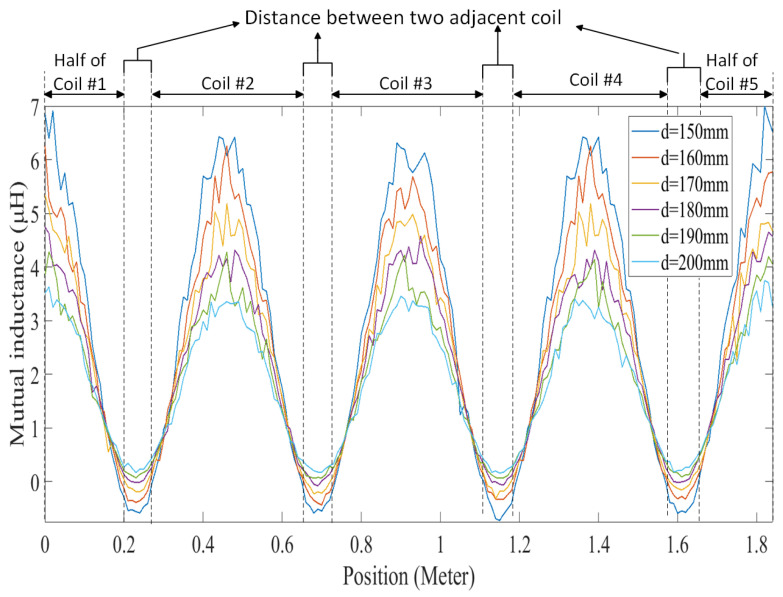
Mutual inductance between the transmitter coils and receiver with different air gaps at various positions.

To acquire the necessary data for predicting mutual inductance, the transmitter and receiver coils were simulated in ANSYS Maxwell. This simulation was conducted to obtain mutual inductance data at various positions for different air gaps. Subsequently, the obtained mutual inductance data were utilized in MATLAB R2022b Simulink to capture current data corresponding to different positions at different vehicle speeds. Figure 9 shows the mutual inductance between the transmitter coils and receiver with different air gaps at various positions. As the receiver moved across the transmitter coils, the mutual inductance reached its maximum when it aligned precisely with each transmitter coil and decreased when the receiver entered the space between adjacent transmitter coils, reaching a minimum at the midpoint between them. This cyclic variation occurred as the receiver crossed each transmitter coil, with the number of cycles corresponding to the number of transmitter coils. In our case, with five transmitter coils, we observed five cycles, resulting in five maxima and minima, as shown in the figure. Importantly, due to the similar structure of the transmitter coils, the maximum and minimum values of mutual inductance remained consistent within each cycle.

Table 3 presents the descriptions and values of the circuit parameters, which were utilized for obtaining the current data in MATLAB Simulink.

**Figure 10:**
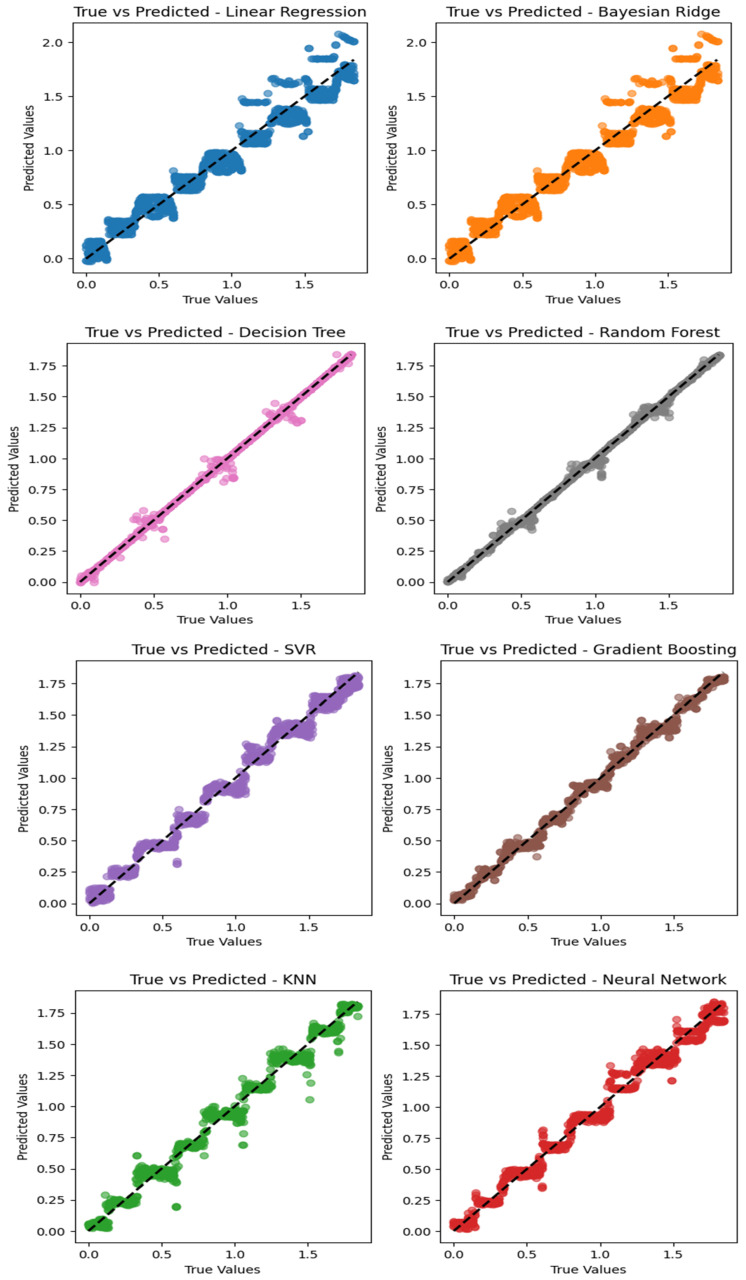
Regression plots of eight different machine learning algorithms in predicting the vehicle’s position.

Figure 10 depicts regression plots illustrating the prediction of the vehicle’s position using eight different machine learning algorithms, namely random forest, decision tree, gradient boosting, KNN, support vector regression, neural network, and Bayesian ridge. Notably, the analysis revealed that random forest exhibited superior performance and accuracy in predicting the actual position, followed closely by the decision tree algorithm, which demonstrated better accuracy compared to the other algorithms in predicting the vehicle’s position. Furthermore, it can be seen that for each algorithm, the largest errors occurred when estimating the position near the center of each coil. This may be due to the small ΔC in these positions, which can otherwise be a strong identifier of the position depending on whether it is a large positive (increasing mutual inductance) or negative (decreasing mutual inductance) value.

**Figure 11:**
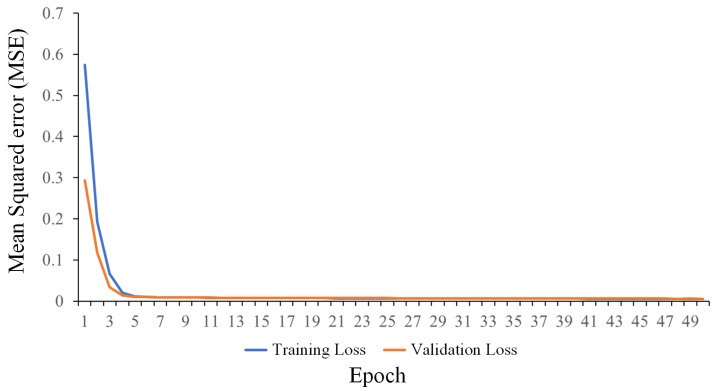
Neural network training per epoch.

Figure 11 shows a graph of the neural network training per epoch. This graph shows that the neural network effectively fitted the data within a few epochs (50 epochs in total). The validation of the training also showed that no overfitting in the neural network occurred. Additionally, although the mean squared error (MSE) settled at around 0.0052, it may be possible to achieve even better generalization (lower MSE) through appropriate tuning and optimization of the hyperparameters and architecture using various methods, as seen in [28].

**Figure 12:**
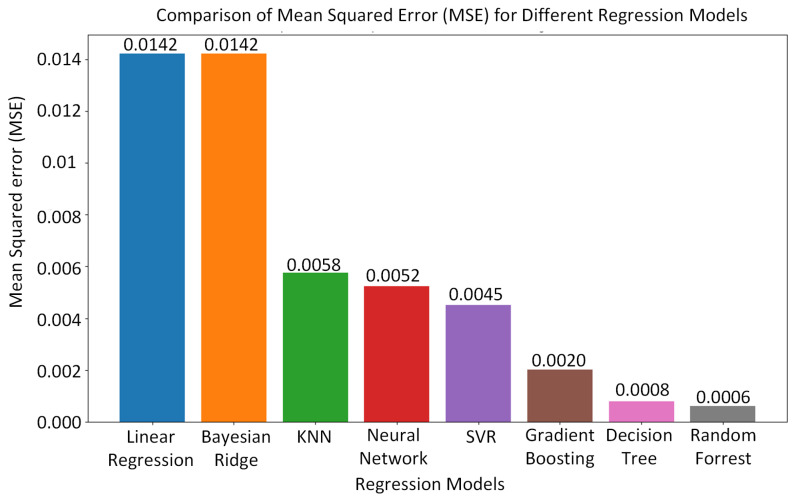
Mean squared error (MSE) for different machine learning algorithms employed for predicting the vehicle’s position.

In Figure 12, the MSE is illustrated for the different machine learning algorithms employed for predicting the vehicle’s position. The random forest algorithm exhibited the lowest MSE, followed closely by the decision tree algorithm, which exhibited a lower MSE compared to the other machine learning algorithms. The random forest algorithm’s superior performance may be due to the fact that it acts as a preventative measure against overfitting and yields more accurate predictions. Moreover, the random forest algorithm introduces extra randomness during tree growth. Instead of solely focusing on the most crucial feature during node splitting, it explores the best feature within a random subset of features. This approach fosters broad diversity, typically leading to superior model performance.

**Figure 13:**
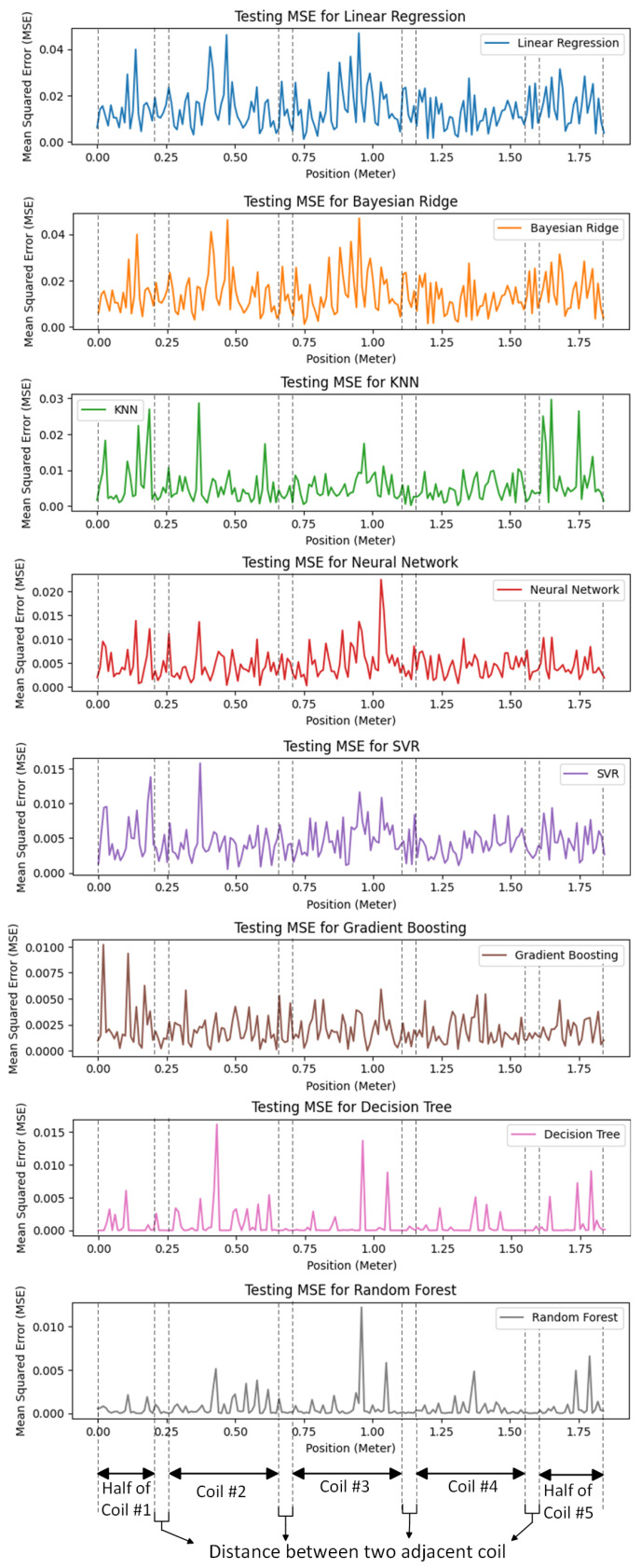
The errors of eight different machine learning algorithms employed for predicting the vehicle’s position at different positions.

Figure 13 illustrates the errors of various machine learning algorithms at different positions. These findings highlight the superior performance of the random forest algorithm, which consistently exhibited the lowest error across different positions, followed by the decision tree algorithm, which demonstrated excellent performance and consistently exhibited very low errors in predicting the vehicle’s position.

**Figure 14:**
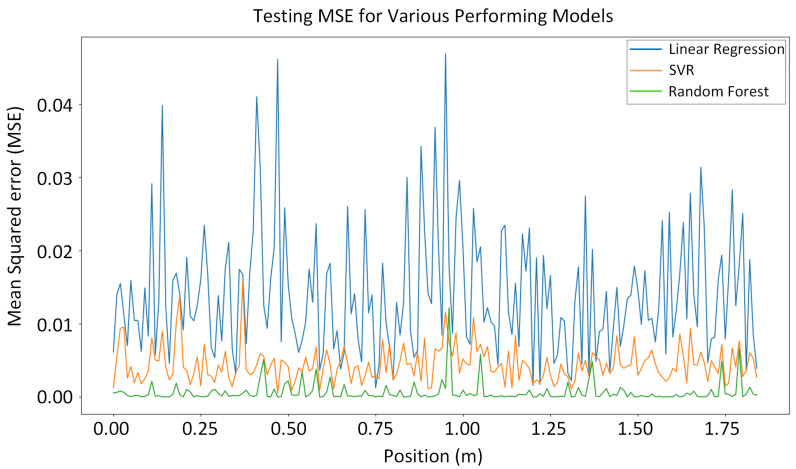
The errors per position of three machine learning algorithms displayed in a single graph.

We chose three machine learning algorithms (random forest, neural network, and SVR) for comparison. Their errors per position are displayed in a single graph, shown in Figure 14. The graph illustrates the superiority of the random forest algorithm in predicting the vehicle’s position with the lowest error.

## 5. Conclusions

Accurate detection of a vehicle’s position is crucial for dynamic wireless charging (DWC) of electric vehicles, ensuring efficient and safe operation. Traditional methods relying on sampling transmitters’ currents become ineffective due to the high speed of vehicles in DWC systems. This paper adopts machine learning algorithms for predicting the vehicle’s position, leveraging their self-learning capability, adaptability to the environment, and swift response. Eight distinct machine learning algorithms are employed for vehicle position detection. Mean squared error bar graphs for each of the eight machine learning algorithms and regression plots for each algorithm demonstrate their accuracy in predicting the actual position. Moreover, this paper illustrates the actual errors of each algorithm for each receiver position, with coil numbers labeled consistently with those in the mutual inductance figure. This aids readers in understanding the ability of each algorithm to predict the position corresponding to each coil or the distance between coils.

Based on the comparison, the ‘tree’-based methods, (decision tree and random forest) demonstrate better generalization with the provided dataset in this application. Finally, the results underscore the superiority of the random forest algorithm in accurately predicting the actual position. Future work will involve incorporating additional real-world parameters for predicting vehicle positions. Furthermore, future work may involve obtaining vehicle position detection data by integrating electric vehicle charging path planning into the existing framework. It should be noted that the results of this research can assist researchers in determining the most accurate machine learning algorithm for detecting vehicle positions in DWC systems. Additionally, it can help engineers in the field adopt the most suitable machine learning algorithm for implementing vehicle position detection systems.

## Figures and Tables

**Table 1:** Transmitter and receiver coupler dimensions.

Parameter	Description	Value
$l_{AL}$	Aluminum plate length	410 mm
$w_{AL}$	Aluminum plate width	410 mm
$l_{fe}$	Ferrite plate length	370 mm
$w_{fe}$	Ferrite plate width	370 mm
$w_{c}$	Coil width	25 mm
$l_{in,coil}$	Inner length of coil	230 mm
$l_{overlap}$	Overlapping length of coils	185 mm
$d_{coil}$	Distance between adjacent transmitter coils	50 mm
d	Air gap	150 mm

**Table 2:** Database for machine learning parameters.

Parameter	Values
Air gap (d)	[150 mm, 160 mm, 170 mm, 180 mm, 190 mm, 200 mm]
Speed (V)	[40 km/h, 50 km/h, 60 km/h, 70 km/h, 80 km/h]
Position (y)	[0, 100 mm, 200 mm, 300 mm, 400 mm, …, 1800 mm]

**Table 3:** Circuit parameters.

Parameter	Description	Value
$V_{in}$	Input voltage	200 V
$L_{p1}$, $L_{p2}$, $L_{p3}$	Self-inductance of transmitter coils	50 μH
$C_{p1}$, $C_{p2},C_{p3}$	Primary series resonant capacitors	70 nF
$L_{2}$	Self-inductance of receiver coil	50 μH
$C_{s}$	Secondary resonant capacitor	70 nH
$C_{b1}$	Input capacitor of buck converter	100 μF
$C_{b2}$	Output capacitor of buck converter	75 μF
$L_{d}$	Inductor of buck converter	9 mH
$f_{s}$	Operating frequency of inverter	85 kHz
f	Frequency of buck converter	20 kHz
$R_{L}$	Load resistor	22 Ω

## Data Availability

The data presented in this study are available on request from the corresponding author. The data are not publicly available due to privacy.
